# Sociodemographic inequalities in vegetables, fruits, and animal source foods consumption in children aged 6–23 months from 91 LMIC

**DOI:** 10.3389/fnut.2023.1046686

**Published:** 2023-02-13

**Authors:** Luiza I. C. Ricardo, Giovanna Gatica-Domínguez, Paulo A. R. Neves, Juliana dos Santos Vaz, Aluisio J. D. Barros, Fernando C. Wehrmeister

**Affiliations:** ^1^International Center for Equity in Health, Federal University of Pelotas, Pelotas, Brazil; ^2^Faculty of Nutrition, Federal University of Pelotas, Pelotas, Brazil

**Keywords:** inequalities, feeding indices, children, low and middle income countries, complementary feeding

## Abstract

**Introduction:**

No multi-country analysis described patterns and inequalities for the brand-new feeding indicators proposed by WHO/UNICEF: zero consumption of vegetables and fruits (ZVF) and consumption of eggs and/or flesh (EFF). Our aim was to describe patterns in the prevalence and social inequalities of ZVF and EFF among children aged 6–23 months in low-and middle-income countries.

**Methods:**

Data from nationally representative surveys (2010–2019) in 91 low-and middle-income countries were used to investigate within-country disparities in ZVF and EFF by place of residence, wealth quintiles, child sex and child age. The slope index of inequality was used to assess socioeconomic inequalities. Analyses were also pooled by World Bank income groups.

**Results:**

The prevalence of ZVF was 44.8% and it was lowest in children from upper-middle income countries, from urban areas, and those 18–23 months. The slope index of inequality showed that socioeconomic inequalities in the prevalence of ZVF were higher among poor children in comparison to richest children (mean SII = −15.3; 95%CI: −18.5; −12.1). Overall, 42.1% of children consumed egg and/or flesh foods. Being a favorable indicator, findings for EFF were generally in the opposite direction than for ZVF. The prevalence was highest in children from upper-middle income countries, from urban areas, and those 18–23 months of age. The slope index of inequality showed pro-rich patterns in most countries (mean SII = 15.4; 95%CI: 12.2; 18.6).

**Discussion:**

Our findings demonstrate that inequalities exist in terms of household wealth, place of residence, and age of the child in the prevalence of the new complementary feeding indicators. Moreover, children from low-and lower-middle countries had the lowest consumption of fruits, vegetables, eggs, and flesh foods. Such findings provide new insights towards effective approaches to tackle the malnutrition burden through optimal feeding practices.

## Introduction

The transition from exclusive breastfeeding to family foods - defined as complementary feeding - covers the period from 6 to 24 months of age, although breastfeeding may continue to 2 years of age and beyond (1). This transition is essential since breast milk alone, after 6 months of age, is no longer sufficient to meet the recommendations for energy, iron and other essential nutrients for optimal growth, body composition, neurodevelopment, strengthening the immune system, and prevention of obesity or noncommunicable diseases in later life (2). The timing of introduction of complementary feeding is also related to physiological and neurodevelopmental maturation, which allows the child to effectively metabolize important nutrients for optimal growth (1, 2).

Healthy complementary feeding practices with early exposure to a variety of flavors and food consistencies, in addition to the flavors provided by breast milk, have positive effects on acceptance of new foods and prevent later feeding problems (1), such as avoidance of fruits and vegetables and obesity (3). Suboptimal complementary feeding practices with insufficient consumption of specific nutrient-rich foods at the age of 6–23 months are associated with nutritional deficiency. For example, poor diets in protein-rich foods (e.g., meat and eggs) are associated with a higher prevalence of stunting and wasting (4, 5). Low consumption of food rich in vitamin A (orange fruits and vegetables) and iron (e.g., meats, and green leafy vegetables) is associated with a higher prevalence of anemia and micronutrient deficiency (5–7).

Indicators for assessing complementary feeding practices of children aged 6 to 23 months were published in 2008 and revised in 2017/2018 by a team of experts commissioned by WHO and UNICEF. Both institutions have concomitantly suggested monitoring the consumption of animal-based protein-rich foods, as well as fruits and vegetables by children within this age range. Information on the consumption of such food types is widely available in surveys of national representativeness, therefore, two new complementary feeding indicators were proposed: egg and/or flesh consumption (EFF) and zero vegetable or fruit consumption (ZVF) (8, 9). The rationale behind these new indicators is that children who consume egg and/or flesh are more likely to attend their needs of specific nutrients important for optimal linear growth, like proteins, vitamin B12, vitamin D, zinc, and others. For vegetables and fruits consumption, the lower is the consumption of these food types the higher are the odds of non-communicable diseases, and the intake early in childhood will influence the optimal later life habits. Although no recommendation has been established for how many servings a day a child over 6 months must have per day, not consuming any food or vegetable serving is deemed as an unhealthy practice (9).

Socioeconomic inequalities in complementary feeding practices in LMICs have previously been documented for core indicators, such as dietary diversity, frequency, and adequacy (10, 11). These studies found a higher prevalence of optimal complementary feeding practices among wealthier children, living in urban areas, mainly in upper middle-income countries. We are unaware of studies that investigated socioeconomic inequalities related to the new EFF and ZVF indicators.

Appropriate diets for everyone, particularly in vulnerable populations, are key goals to achieve two of the Sustainable Development Goals for 2030, zero hunger and good health and well-being (12). The new complementary feeding indicators regarding animal protein and vegetable/fruit consumption can provide new insights into how to tackle inappropriate feeding choices that can negatively affect children’s health in LMICs. In the present study, we describe socioeconomic inequalities in the prevalence of EFF and ZVF consumption among children between 6 and 23 months of age in LMICs.

## Materials and methods

This is a multi-country study based on nationally representative cross-sectional surveys. We analyzed the latest health survey since 2010 (median year = 2017), from 91 LMICs: 45 Demographic Health Surveys (DHS; https://dhsprogram.com/), 45 Multiple Indicator Cluster Survey (MICS; http://mics.unicef.org/), and one modified version of DHS from Bolivia (*Encuesta de Demografía y Salud* 2016). These household surveys are carried out every 3 or 5 years to monitor the health and nutrition of the country population. The methodology of these surveys is comparable in terms of questionnaires, field work, and sampling design (13). The sampling is based on a stratified two-stage cluster design with enumeration areas in the first stage and households selected in the sampled enumeration areas. In the selected households, a questionnaire on feeding practices of their youngest child was applied to mothers of children aged 6–23 months.

### Feeding indicators

Two new recommended Infant and Young Child Feeding (IYCF) indicators were estimated for children aged 6–23 months based on food consumption in the 24 h preceding the survey: (1) zero vegetable or fruit consumption (ZVF) – the percentage of children who did not consume any vegetables or fruits; and (2) any egg or flesh food consumption (EFF) – the percentage of children who consumed egg or flesh food in any quantity (14). These indicators are based on standardized questions. ZVF is based on consumption of vitamin A-rich fruits and vegetables, as well as other fruits and vegetables. Plantains and starchy roots and tubers do not count for this indicator.

### Sociodemographic variables

DHS and MICS datasets have available the household asset scores. Household assets such as building materials, infrastructure (e.g., water and electricity), and assets (presence of car, television, radio, etc.) were analyzed to generate the household asset score adjusted for urban or rural residence according to the methodology developed by DHS (15). Based on this asset score, households were classified into wealth quintiles, where the first quintile represents the 20% poorest households and the fifth quintile the 20% richest households. For the survey considered as a modified version of DHS, we had to calculate the household asset index using the exact same DHS methodology to make it comparable to the DHS and MICS surveys.

### Statistical analysis

ZVF and EFF prevalence were described by country, age groups (i.e., 6–11, 12–17, and 18–23 months), place of residence (urban or rural), wealth and sex of the child (female or male). Countries were grouped according to the World Bank income group classifications for the year of the survey (16). We used the population of children within 6–23 months of age in each country to weight the pooled income group estimates, retrieved from the World Bank Population Estimates.

As a measure of absolute socioeconomic inequalities, we calculated the slope index of inequality (SII) in each country. This index is derived through a logistic regression model, taking the ZVF or EFF prevalence as the outcome and the wealth quintiles as the independent variable. The SII represents the absolute difference in the fitted value of ZVF or EFF prevalence between the highest and the lowest values of the wealth index scale taking the intermediate values into account (17). SII values for prevalence range from −100 to +100, with zero representing no inequalities across the wealth scale; positive values represent a pro-rich distribution (higher prevalence among richer quintiles) and negative values a pro-poor distribution (higher prevalence among poorest quintiles).

Urban and rural residence was based on the classification of the sampled clusters adopted by the national governments at the time of the survey. We graphed dropline charts from the SII to improve visualization of socioeconomic inequalities by each country for both feeding indicators. We used Equiplots to assess urban–rural inequalities, and gap size within countries were presented in decreasing order. Equiplots are dot line graphs where the prevalence in each group and the distance between groups represents absolute inequality. *p*-values < 0.05 were considered statistically significant. All analysis were performed in Stata 17 (StataCorp, College Station, TX, United States). The data from national surveys analyzed in the present study is publicly available, and the institutions that conducted the surveys in each country dealt with the ethical clearance.

## Results

Of the 91 surveys analyzed, 26 were from low-income countries (LIC), 37 from lower-middle (LMIC) and 28 upper-middle-income countries (UMIC), representing 83.9% of all low-income countries, 71.2% of all lower-middle, and 50% of all upper-middle-income countries in the world as of 2017, the median year of the surveys used.

### Zero consumption of vegetables and fruits

**Figure 1:**
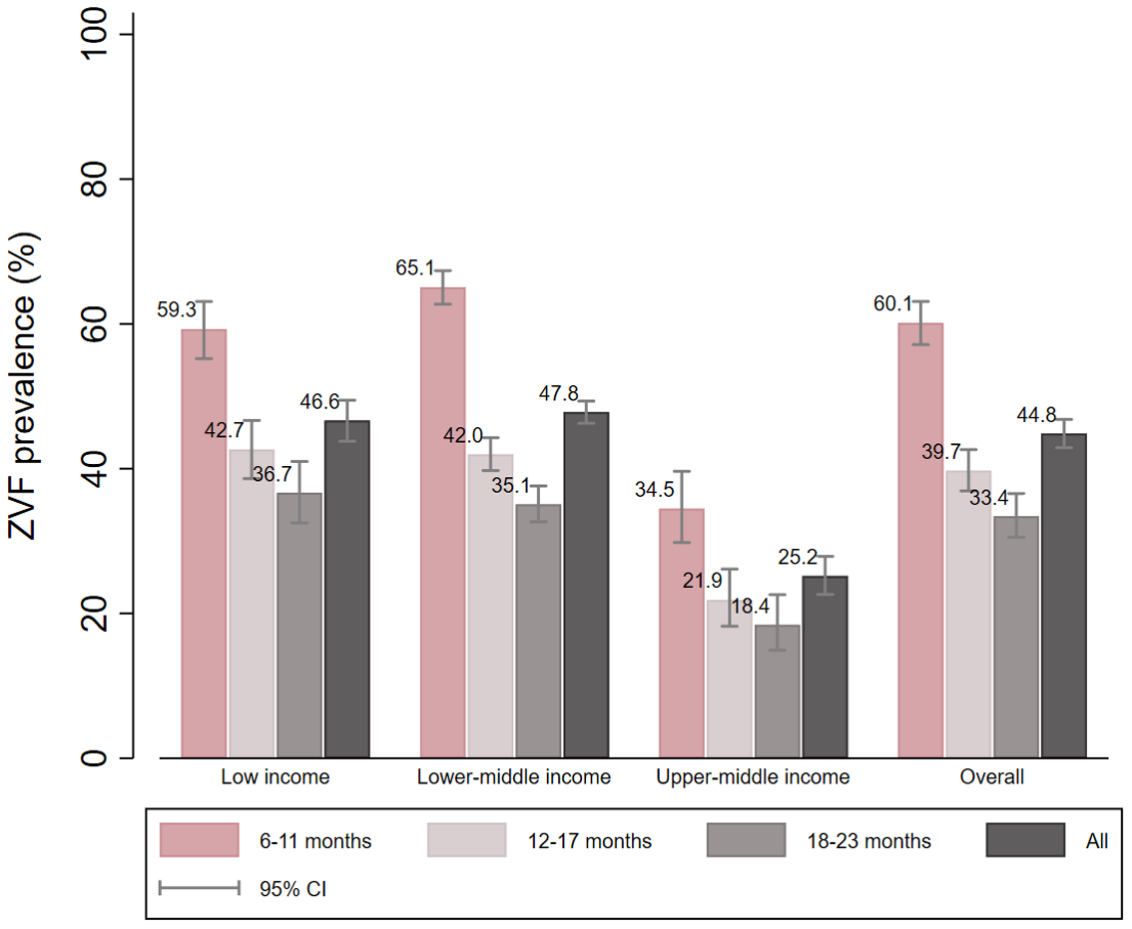
ZVF prevalence according to country income and child age groups.

The prevalence of ZVF according to country income classification and age group is shown in Figure 1. The overall ZVF prevalence was 44.8%, being similar in low-and lower-middle-income countries (LIC 46.6, 95% CI: 43.8 to 49.5%, and LMIC 47.8, 95% CI: 46.3 to 49.4), and significantly lower in upper-middle income countries (25.2, 95% CI: 22.6 to 27.9). Across all country income groups ZVF prevalence declined with age, being substantially higher in 6–11 months children both in the pooled analysis and by country income group.

**Figure 2:**
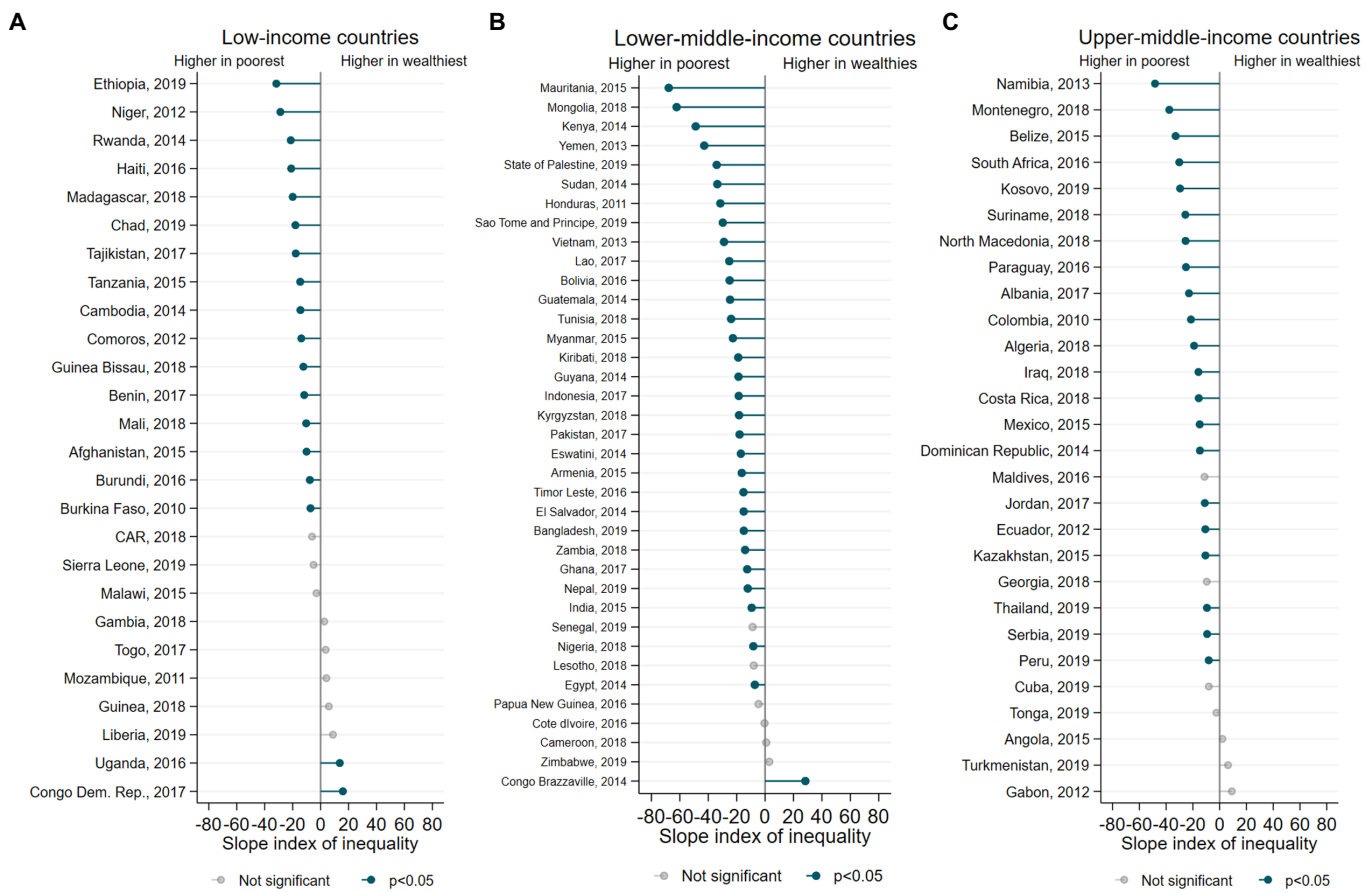
**(A-C)** Inequalities in the ZVF prevalence according to wealth quintiles.

The SII results for ZVF prevalence according to wealth quintiles is shown in Figure 2. For most countries the ZVF prevalence among the poorest was higher than in the richest group (negative SII values) for all income groups. Also, wider income inequalities were found for lower-middle-income countries. On the other hand, only 3 countries presented a significantly higher ZVF prevalence in the wealthiest groups (Congo Dem. Rep., Uganda, and Congo Brazzaville).

Supplementary Table 1 shows overall prevalence of ZVF and stratified by age groups and child sex categories. The prevalence of ZVF ranged from 2.5% in Serbia to 74.9% in Burkina Faso. On average, ZVF prevalence declined with age, from 48.1% in 6–11 months old children to 24.4% in 18–23 months old children. The ZVF prevalence among girls ranged from 2.2% in Serbia to 74.8% in Burkina Faso, while among boys ranged from 2.8% to 74.9, in the same countries.

The prevalence of ZVF according to area of residence (urban or rural) is shown in Supplementary Figure 1. Overall, the ZVF prevalence was higher in children from rural areas. The mean difference between urban and rural area in ZVF prevalence was 7.5 p.p., ranging from 0.2 p.p. in Egypt to 36 p.p. in Mauritania.

### Egg and/or flesh consumption

**Figure 3:**
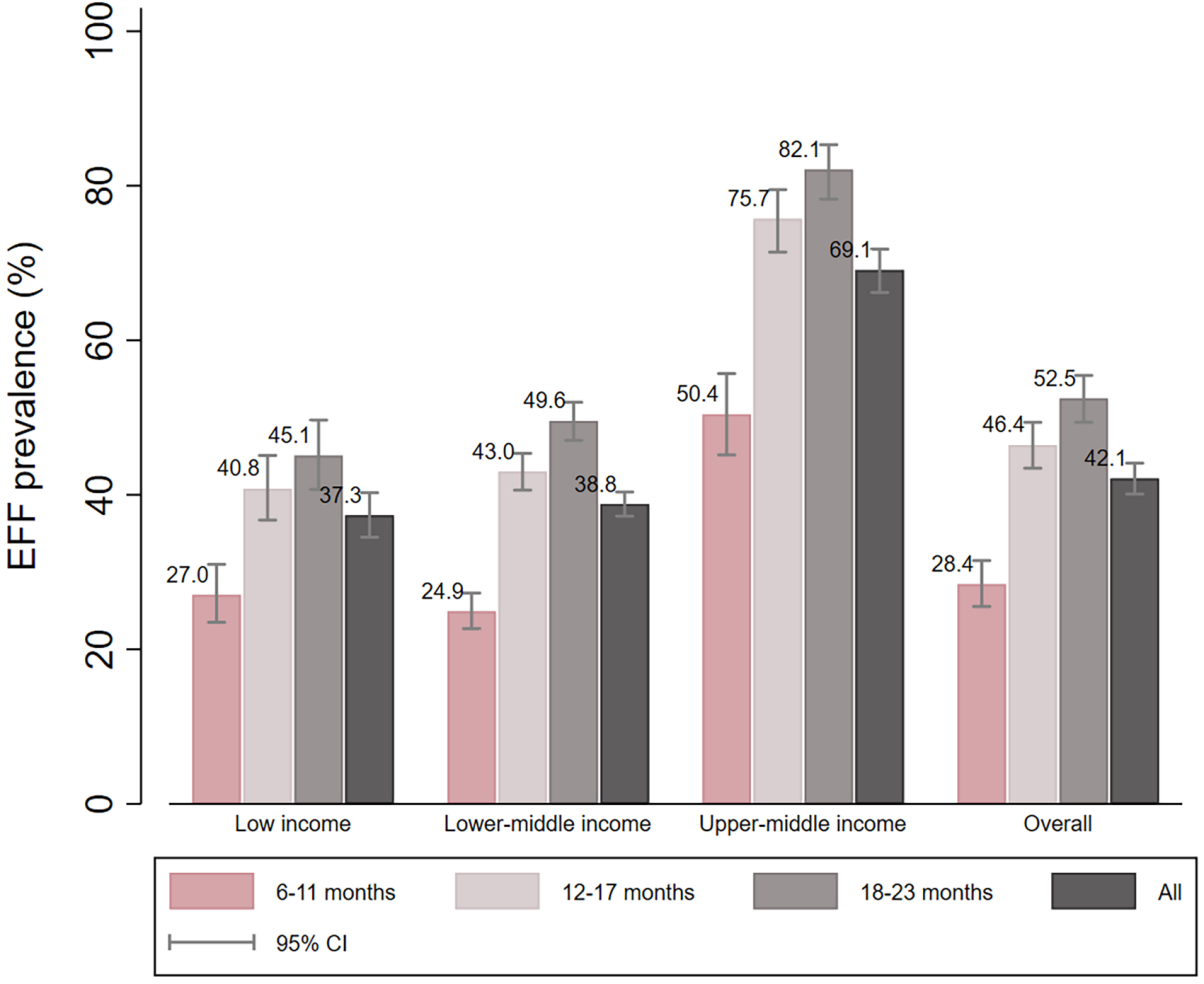
EFF prevalence according to country income and child age groups.

The overall EFF prevalence was 42.1%, and the highest prevalence was found for upper-middle income countries (69.1, 95% CI: 66.2 to 71.8). Regarding age groups, EFF prevalence increased with age, being lowest in 6–11 months children and highest in 18–23 months children, both in the pooled analysis and by country income group. The youngest age group (6–11 months) presented disproportionally lower EFF prevalence in all income groups (Figure 3).

**Figure 4:**
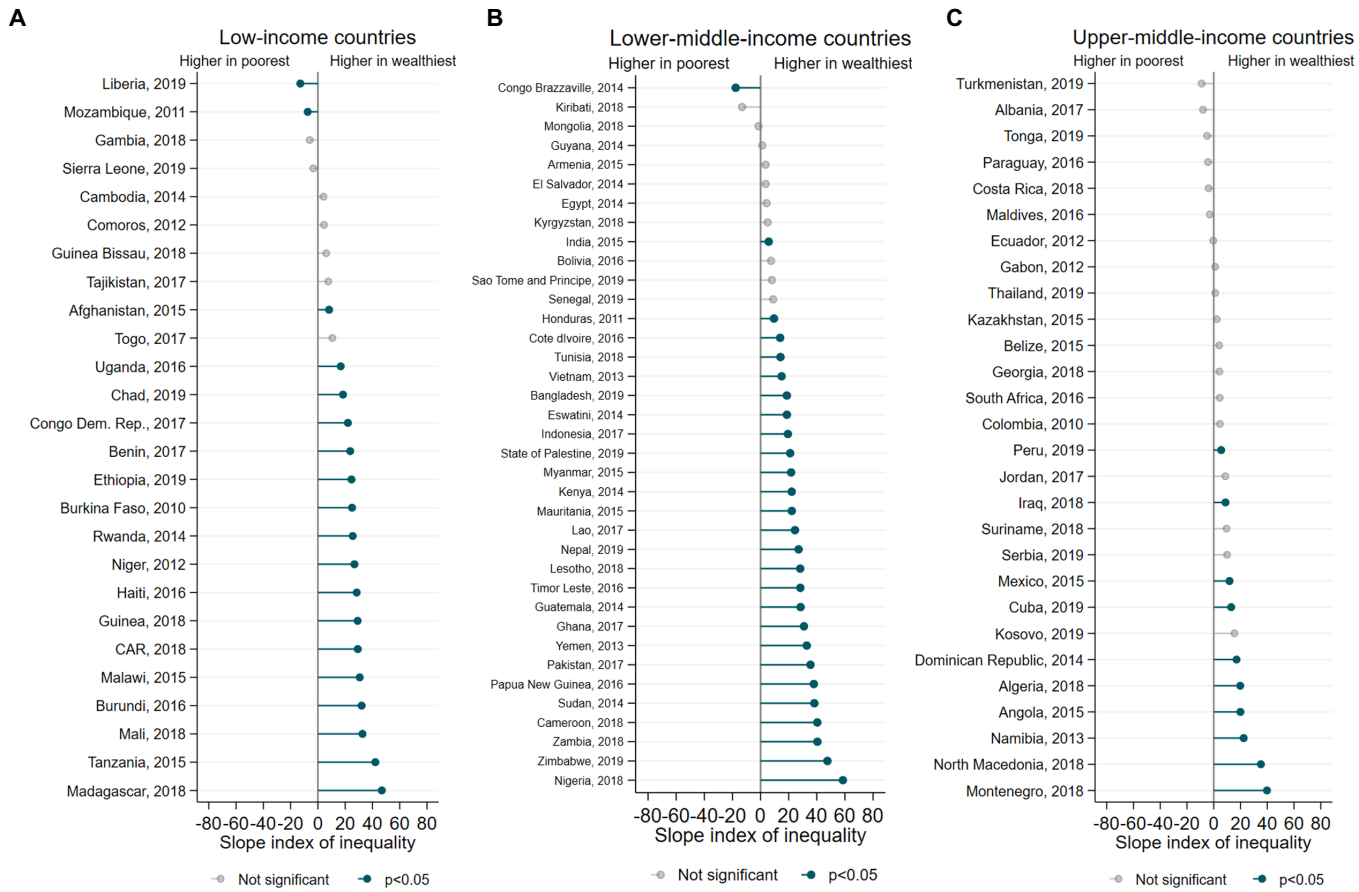
**(A-C)** Inequalities in the EFF prevalence according to wealth quintiles.

Figure 4 presents the SII for the EFF prevalence according to wealth quintiles. We found positive SII values for most countries, representing a higher EFF prevalence among the wealthiest groups. Three countries presented negative SII results, with a higher prevalence of EFF among the poorest (Liberia, Mozambique, and Congo Brazzaville).

The overall prevalence of EFF and stratified by age group and sex of the child is shown in Supplementary Table 2. The highest overall prevalence of EFF was in Peru (Overall: 91.6%; Girls: 92.1%, Boys: 91.0%) while the lowest prevalence was found in Niger (Overall: 16.8%; Girls: 16.4%, Boys: 17.2%). On the other hand, EFF prevalence increased with age, being on average 38.8% in 6–11 months old children and 72.2% in 18–23 months old children. Taking all countries into consideration, boys had a slightly higher prevalence of EFF than girls (0.3 p.p. of difference), on average.

Supplementary Figure 2 shows the EFF prevalence according to area of residence. The prevalence of EFF was higher among children living in urban areas for most countries, with 8 p.p. of mean difference between rural and urban areas, ranging from 0.1 p.p. in Thailand to 27 p.p in Niger.

## Discussion

As far as we know, this is the first paper to report the prevalence of two new diet quality indicators, namely ZVF and EFF, in infants and young children from 91 LMICs, and describes the patterns in their prevalence according to two dimensions of inequality (i.e., socioeconomic and demographic). The results of our study showed that among all countries included, 44.8% of the children presented zero consumption of vegetables and fruits, while 42.1% consumed egg and/or flesh foods. Additionally, our findings show inequalities in terms of household wealth, place of residence, and age of the child, with the poorest, rural, and youngest children being at disadvantage for both indicators. In a previous publication in 33 countries from Sub-Saharan Africa, the determinants of ZVF and EFF in children aged 6–23 months of age were mapped, showing that younger children, living in rural areas, and from uneducated families were the most disadvantaged groups for both feeding indicators (18).

Optimal complementary feeding practices have a direct effect on the health and nutritional status of children. However, in many LMICs, children are not given the necessary foods to thrive. Therefore, monitoring such feeding practices in a thorough way is part of the effort to tackle the burden of suboptimal complementary feeding practices, especially in poor-resource settings. For this reason, the WHO and UNICEF improved and developed new infant and young child feeding indicators. However, specifically, the two new feeding indicators from this study may have a few flaws that should be taken into consideration when used. For example, because one indicator shows a negative behavior (ZFV) and the other a positive behavior (EFF), the interpretation can probably be confusing and misinterpreted, especially if they are studied together. Likewise, presenting an overall prevalence of 6 to 23 months can mask inappropriate practice at some ages, so the presentation of the stratified prevalence by age groups makes more sense due to the gradual introduction of family foods that is usually practiced in families.

Regarding the prevalence of ZVF, we observed an inversely related inequality for the country income group, whereas the upper middle-income countries presented the lowest prevalence compared to the low-and lower middle-income countries. On the contrary, the prevalence of EFF was higher in countries of upper-middle income compared to the other two income groups. Similar patterns were observed in previous studies in LMICs (11, 19, 20). An analysis of availability and cost of fruits and vegetables in 18 LMICs showed that the low consumption of fruits and vegetables was associated with low affordability, particularly in individuals of LIC (19). The prevalence of animal-source food consumption showed a pronounced between-country wealth inequality in children of 80 LIMCs (11). A recent analysis of 185 countries revealed a wide-ranging disparity of daily servings of animal-source foods in children, with a high consumption of processed meat which is affordable but poor in micronutrients (20).

Our findings showed improvements in both indicators studied (i.e., increasing consumption of eggs and protein-rich foods and decline in lack of vegetables and fruits consumption) as the age of the child increased. This is in accordance with the literature on diet diversity, frequency and adequacy (21). However, our findings were particularly alarming for children aged 6–11 months, mainly in low-and lower-middle income countries. The negative results found for the youngest age group might indicate inadequate quality and timing of complementary feeding practices (22). For example, in our study the EFF prevalence in low-and lower-income countries was around 25% while in upper-middle countries was around 50%. It is known that there has been an increase in the practice of baby-led weaning (BLW) in higher income countries (23), which may explain the higher EFF prevalence and the smaller ZVF prevalence observed in upper-middle income countries (24). The BLW method is a feeding approach in which children from 6 months of age are exposed to the family meals, including solid foods such as eggs and flesh foods. The aforementioned can be supported with the findings of a study where children aged 6–23 months from upper-middle income countries had a higher minimum dietary diversity compared with their counterparts from low-and lower-middle income countries (11). Appropriate complementary feeding is recommended to start at 6 months, when the infants’ needs for energy and nutrients begins to outrun breastmilk alone (25) at an early age.

For most countries regardless of the income group, the ZVF prevalence was higher among the poorest children, whereas a higher EFF prevalence among the wealthiest was observed. These results show a pro-rich inequality, which is consistent with evidence from 80 LMIC where the consumption of other fruits and vegetables (not vitamin A-rich fruits and vegetables), as well as animal-source was significantly higher among the wealthiest children compared with their counterparts (SII = 6.4 and 6.6, respectively), most likely due to the high cost of these type of foods (11).

On the other hand, our results showed that poorest children from Congo Democratic Republic and Uganda presented a significantly higher prevalence of fruits and vegetables consumption compared with their counterparts. Additionally, poorest children from Liberia and Mozambique had a significantly higher prevalence of any eggs and/or flesh food consumption compared with the wealthiest children. Congo Brazzaville was the only country where the poorest children had a significant higher consumption of both indicators studied compared with their wealthiest counterparts. Two hypothesis that may explain this phenomenon could be that in these African countries (1) policies, national strategies and agricultural interventions were implemented to address the lack of food availability due to environmental conditions to improve simultaneously nutrition outcomes, especially among the most poorest families thus improving their food security (e.g., Nutrition Smart Agriculture) (26); and/or (2) children may be experiencing a nutritional transition and the wealthiest children could be more exposed to an unhealthy diets (i.e., low consumption of fruits and vegetables, as well as eggs and/or flesh foods) than the poorest children (27). There is evidence that in some African subregions childhood overweight has increased overtime. For example, in Liberia and Mozambique childhood overweight has increased since 2012, whereas stunting prevalence has decreased during this period (28, 29).

Our study has some limitations that need to be considered. The consumption of fruits and vegetables in some countries may be strongly influenced by its availability, driven by seasonality and culture patterns; the list-based questionnaire for foods consumed in the previous 24 h does not capture the child’s food consumption pattern comprehensively, especially when influenced by availability-related factors, and, also, recall bias cannot be disregarded when working with such questionnaire; the indicator any eggs and/or flesh foods does not consider the food quantity consumed which is also relevant for children in a rapid growth period; data for countries with a large population of children under 2-years old is lacking, like Brazil and China. On the other hand, this is the first multicountry study describing different inequality patterns in the new feeding indicators recommended by the WHO and UNICEF; the generalization of the findings is enhanced due to the diverse geographical span of the surveys included; the high compatibility between DHS and MICS surveys allowed the pooled investigation of several LMICs; and, the WHO and UNICEF acknowledged the usage of DHS/MICS surveys as an important data source to investigate feeding patterns among children.

In conclusion, our study presented national and grouped estimates of ZVF and EFF prevalence, indicating inequalities according to household wealth, place of residence, and age of the child for both indicators of child complementary feeding. Our results highlight the relevance to develop nutrition policies and key multi-sectorial strategies to implement direct and, mainly, indirect interventions and programs which consider the context of each country to make fruit, vegetables, and animal-source foods such as eggs and flesh foods available, accessible and, above all, affordable, especially for children under 2 years of age for being within the 1,000-days window. Also, national policies must restrict the access to ultra-processed foods, which are unhealthy and cheaper, and thus prevent the most vulnerable population from consuming them as a substitute for fruit and vegetables *in natura* and fresh animal-source foods. In this sense, the development of complementary feeding guidelines at the country level is critical for the population to have evidence-based information guide on the adequate feeding practices in infants, but taking into account the context of each country, such as culture. On another note, the COVID-19 pandemic has worsened the food insecurity in LMIC putting at risk the intake of adequate nutrients for the optimal growth of these children since the combined effects of COVID-19, the mitigation measures, and the emerging global recession, without large-scale coordinated action, have disrupted the functioning of food systems and impacted on diets by having to cope with unhealthy foods (30, 31). Therefore, it is essential to continue monitoring these indicators at the national level and carrying out analysis of inequalities to identify subpopulations with the greatest vulnerability and thus the decision makers of the countries redesign policies and target programs and interventions accordingly.

## Data availability statement

Publicly available datasets were analyzed in this study. This data can be found at: DHS: https://dhsprogram.com/data/available-datasets.cfm, MICS: https://mics.unicef.org/surveys.

## Author contributions

LR and GG-D conceived this work and participated in the design, writing, and interpretation of the data. LR performed data analysis. GG-D, PN, JV, AB, and FW participated in writing and revising the paper. All authors contributed to the article and approved the submitted version.

## Funding

This work was supported by the Bill & Melinda Gates Foundation (grant no. INV-010051/OPP1199234), Wellcome Trust (grant no. 101815/Z/13/Z), and ABRASCO.

## Conflict of interest

The authors declare that the research was conducted in the absence of any commercial or financial relationships that could be construed as a potential conflict of interest.

## Publisher’s note

All claims expressed in this article are solely those of the authors and do not necessarily represent those of their affiliated organizations, or those of the publisher, the editors and the reviewers. Any product that may be evaluated in this article, or claim that may be made by its manufacturer, is not guaranteed or endorsed by the publisher.

## References

[1] Przyrembel H. Timing of introduction of complementary food: short-and long-term health consequences. Ann Nutr Metab. (2012) 60:8–20. doi: 10.1159/000336287 22555185

[2] Campoy C Campos D Cerdó T Diéguez E Garciá-Santos JA. Complementary feeding in developed countries: the 3 Ws (when, what, and why?). Ann Nutr Metab. (2018) 73:27–36. doi: 10.1159/000490086, PMID: 30196294

[3] Northstone K Emmett P Nethersole F. The effect of age of introduction to lumpy solids on foods eaten and reported feeding difficulties at 6 and 15 months. J Hum Nutr Diet. (2001) 14:43–54. doi: 10.1046/j.1365-277X.2001.00264.x, PMID: 11301932

[4] Headey DD Palloni G. Stunting and wasting among Indian preschoolers have moderate but significant associations with the vegetarian status of their mothers. J Nutr. (2020) 150:1579–89. doi: 10.1093/jn/nxaa042, PMID: 32171005 PMC7269725

[5] Mya KS Kyaw AT Tun T. Feeding practices and nutritional status of children age 6-23 months in Myanmar: a secondary analysis of the 2015-16 demographic and health survey. PLoS One. (2019) 14:e0209044. doi: 10.1371/journal.pone.0209044, PMID: 30601848 PMC6314612

[6] Donkor WES Adu-Afarwuah S Wegmüller R Bentil H Petry N Rohner F. Complementary feeding indicators in relation to micronutrient status of Ghanaian children aged 6–23 months: results from a National Survey. Life. (2021) 11:969. doi: 10.3390/life11090969 34575118 PMC8468967

[7] Karlsson O Kim R Hasman A Subramanian SV. Consumption of vitamin-A-rich foods and vitamin a supplementation for children under two years old in 51 low-and middle-income countries. Nutrients. (2022) 14:188. doi: 10.3390/nu14010188 PMC8747127 35011064

[8] USAID. STAT compiler. The DHS. Program. (2022).

[9] World Health Organization and the United Nations Children’s Fund 2021 Indicators for assessing infant and young child feeding practices. Vol. WHA55 A55/. World Health Organization and the United Nations Children’s Fund (UNICEF). Geneva; 19.

[10] White JM Bégin F Kumapley R Murray C Krasevec J. Complementary feeding practices: current global and regional estimates. Matern Child Nutr. (2017) 13:e12505. doi: 10.1111/mcn.12505 29032623 PMC6865887

[11] Gatica-Domínguez G Neves PAR Barros AJD Victora CG. Complementary feeding practices in 80 low-and middle-income countries: prevalence of and socioeconomic inequalities in dietary diversity, meal frequency, and dietary adequacy. J Nutr. (2021) 151:1956–64. doi: 10.1093/jn/nxab088, PMID: 33847352 PMC8245881

[12] United Nations. The sustainable development goals report. New York, USA: United Nations Publications. (2018) 1–56.

[13] Hancioglu A Arnold F. Measuring coverage in MNCH: tracking Progress in health for women and children using DHS and MICS household surveys. PLoS Med. (2013) 10:e1001391. doi: 10.1371/journal.pmed.1001391, PMID: 23667333 PMC3646216

[14] WHO 2021 World Health Organization and the United Nations Children’s fund (UNICEF). Indicators for assessing infant and young child feeding practices: Definitions and measurement methods. Geneva.

[15] Rutstein SO 2015 Steps to constructing the new DHS wealth index. Vol. Demographi, Usaid.

[16] World Bank Country and Lending Groups–World Bank Data Help Desk (2023) Available at: https://datahelpdesk.worldbank.org/knowledgebase/articles/906519-world-bank-country-and-lending-groups (Accessed January 31, 2023).

[17] Barros AJD Victora CG. Measuring coverage in MNCH: determining and interpreting inequalities in coverage of maternal, newborn, and child health interventions. Madise N, editor. PLoS Med. (2013) 10:e1001390. doi: 10.1371/journal.pmed.1001390 23667332 PMC3646214

[18] Hailu BA Geremew BM Liverani S Abera KS Beyene J Miheretu BA. Mapping and determinants of consumption of egg and/or flesh foods and zero vegetables or fruits among young children in SSA. Sci Rep. (2022) 12:11924. doi: 10.1038/s41598-022-15102-z, PMID: 35831382 PMC9279389

[19] Miller V Yusuf S Chow CK Dehghan M Corsi DJ Lock K. Availability, affordability, and consumption of fruits and vegetables in 18 countries across income levels: findings from the prospective urban rural epidemiology (PURE) study. Lancet Glob Health. (2016) 4:e695–703. doi: 10.1016/S2214-109X(16)30186-3, PMID: 27567348

[20] Miller V Reedy J Cudhea F Zhang J Shi P Erndt-Marino J. Global, regional, and national consumption of animal-source foods between 1990 and 2018: findings from the global dietary database. Lancet Planet Health. (2022) 6:e243–56. doi: 10.1016/S2542-5196(21)00352-1, PMID: 35278390 PMC8926870

[21] Patrick H Nicklas TA. A review of family and social determinants of Children’s eating patterns and diet quality. 2013; 24: 83–92. 10.1080/07315724.2005.10719448 15798074

[22] Choudhury S Headey DD Masters WA. First foods: diet quality among infants aged 6–23 months in 42 countries. Food Policy. (2019) 88:101762. doi: 10.1016/j.foodpol.2019.101762, PMID: 31853163 PMC6894322

[23] Langley-Evans SC. Complementary feeding: should baby be leading the way? J Hum Nutr Diet. (2022) 35:247–9. doi: 10.1111/jhn.12988, PMID: 35066946 PMC9303566

[24] Rowan H Lee M Brown A. Differences in dietary composition between infants introduced to complementary foods using baby-led weaning and traditional spoon feeding. J Hum Nutr Diet. (2019) 32:11–20. doi: 10.1111/jhn.12616, PMID: 30585361

[25] Complementary feeding (2022). Available at: https://www.who.int/health-topics/complementary-feeding#tab=tab_1 (Accessed January 31, 2023).

[26] Nutrition Smart Agriculture in the Democratic Republic of Congo (2020) Available at: https://documents.worldbank.org/en/publication/documents-reports/documentdetail/875971596177465224/nutrition-smart-agriculture-in-the-democratic-republic-of-congo (Accessed January 31, 2023).

[27] Victora CG Joseph G Silva ICM Maia FS Vaughan JP Barros FC. The inverse equity hypothesis: analyses of institutional deliveries in 286 national surveys. Am J Public Health. (2018) 108:464–71. doi: 10.2105/AJPH.2017.304277 29470118 PMC5844402

[28] Africa – Regional Overview of Food Security and Nutrition. Africa–regional overview of food security and nutrition 2021 (2021). 2021 p.

[29] UNICEF/WHO/The World Bank Group 2021 Levels and trends in child malnutrition: UNICEF/WHO/The World Bank Group joint child malnutrition estimates: key findings of the edition. Available at: https://www.who.int/publications/i/item/9789240025257 (Accessed January 31, 2023).

[30] Laborde D Martin W Vos R. Impacts of COVID-19 on global poverty, food security, and diets: insights from global model scenario analysis. Agric Econ. (2021) 52:375–90. doi: 10.1111/agec.12624, PMID: 34230728 PMC8251321

[31] UNSDG (Policy brief: The impact of COVID-19 on food security and nutrition) (2020) [internet]. Available at: https://unsdg.un.org/resources/policy-brief-impact-covid-19-food-security-and-nutrition (Accessed January 31, 2023).

