# Supplementary material for: Sociodemographic inequalities in vegetables, fruits, and animal source foods consumption in children aged 6–23 months from 91 LMIC
Source: Front Nutr. 2023 Feb 13;10:1046686. doi: 10.3389/fnut.2023.1046686 (PMC9972219; doi:10.3389/fnut.2023.1046686)
Supplement: Supplementary file 1 [file Data_Sheet_1.docx]

**Supplementary Material**

**Supplementary Table 1:** Prevalence of ZVF according to child age group and sex

|  |  |  |  |  |  |  |  |  |  |  |  |  |  |  |  |  |
| --- | --- | --- | --- | --- | --- | --- | --- | --- | --- | --- | --- | --- | --- | --- | --- | --- |
|  |  |  |  |  |  |  |  |  |  |  |  |  |  |  |  |  |
|  | **Zero vegetable or fruit** | | | | | | | | | | | | | | | |
|  | **Age groups** | | | | | | | | | |  | **Child sex** | | | | |
| **Survey** | **Overall** |  | **6-11** | |  | **12-17** | |  | **18-23** | |  | **Girls** | |  | **Boys** | |
|  | **%** |  | **N** | **%** |  | **N** | **%** |  | **N** | **%** |  | **N** | **%** |  | **N** | **%** |
| **Low income countries** |  |  |  |  |  |  |  |  |  |  |  |  |  |  |  |  |
| Afghanistan, 2015 | 59.7 |  | 2627 | 69.4 |  | 3599 | 55.1 |  | 1852 | 54.3 |  | 4152 | 57.9 |  | 3926 | 61.5 |
| Benin, 2017 | 57.8 |  | 1370 | 22.8 |  | 1309 | 12.4 |  | 1179 | 11.1 |  | 1986 | 59.4 |  | 1898 | 56.3 |
| Burkina Faso, 2010 | 74.9 |  | 1482 | 74.1 |  | 1187 | 51.7 |  | 1215 | 43.9 |  | 2092 | 74.8 |  | 2056 | 74.9 |
| Burundi, 2016 | 15.7 |  | 1435 | 88.1 |  | 1445 | 70.8 |  | 1268 | 64.4 |  | 1964 | 14.8 |  | 1894 | 16.6 |
| Central African Republic, 2018 | 39.1 |  | 839 | 49.8 |  | 949 | 35.3 |  | 739 | 31.4 |  | 1260 | 40.6 |  | 1267 | 37.5 |
| Cambodia, 2014 | 35.4 |  | 2248 | 40.4 |  | 2326 | 24.2 |  | 1925 | 19.5 |  | 1073 | 36.5 |  | 1054 | 34.3 |
| Chad, 2019 | 48.9 |  | 315 | 61.4 |  | 311 | 51.8 |  | 243 | 47.2 |  | 2763 | 49.6 |  | 2683 | 48.3 |
| Comoros, 2012 | 53.9 |  | 501 | 77.1 |  | 548 | 69.9 |  | 416 | 60.0 |  | 436 | 47.0 |  | 433 | 60.3 |
| Congo Dem. Republic, 2017 | 28.4 |  | 588 | 83.9 |  | 883 | 60.3 |  | 438 | 40.0 |  | 3294 | 31.0 |  | 3205 | 25.6 |
| Ethiopia, 2019 | 69.3 |  | 829 | 85.6 |  | 1106 | 57.9 |  | 789 | 46.7 |  | 747 | 68.4 |  | 718 | 70.1 |
| Gambia, 2018 | 63.4 |  | 784 | 90.7 |  | 669 | 64.7 |  | 740 | 52.6 |  | 1385 | 63.8 |  | 1339 | 63.0 |
| Guinea, 2018 | 62.8 |  | 556 | 61.7 |  | 625 | 54.6 |  | 471 | 46.4 |  | 987 | 63.9 |  | 922 | 61.8 |
| Guinea Bissau, 2018 | 69.4 |  | 748 | 56.1 |  | 655 | 28.6 |  | 724 | 20.4 |  | 1096 | 69.2 |  | 1097 | 69.7 |
| Haiti, 2016 | 54.7 |  | 558 | 74.7 |  | 511 | 44.4 |  | 454 | 43.7 |  | 832 | 53.8 |  | 820 | 55.5 |
| Liberia, 2019 | 55.8 |  | 1269 | 36.2 |  | 1411 | 24.1 |  | 1179 | 21.8 |  | 759 | 56.8 |  | 764 | 54.7 |
| Madagascar, 2018 | 27.4 |  | 902 | 77.3 |  | 1016 | 45.7 |  | 795 | 38.5 |  | 1962 | 28.8 |  | 1897 | 26.0 |
| Malawi, 2015 | 23.2 |  | 1168 | 53.3 |  | 1157 | 29.3 |  | 955 | 23.1 |  | 2430 | 22.7 |  | 2317 | 23.7 |
| Mali, 2018 | 54.0 |  | 1627 | 37.5 |  | 1627 | 17.1 |  | 1493 | 14.0 |  | 1401 | 53.0 |  | 1312 | 54.9 |
| Mozambique, 2011 | 36.1 |  | 1209 | 80.8 |  | 1251 | 62.3 |  | 800 | 55.4 |  | 1580 | 35.4 |  | 1700 | 36.9 |
| Niger, 2012 | 67.5 |  | 888 | 37.8 |  | 765 | 18.8 |  | 701 | 16.7 |  | 1621 | 65.4 |  | 1639 | 69.5 |
| Rwanda, 2014 | 25.2 |  | 902 | 75.3 |  | 1040 | 43.1 |  | 701 | 33.9 |  | 1175 | 25.1 |  | 1179 | 25.4 |
| Sierra Leone, 2019 | 51.8 |  | 1836 | 60.1 |  | 2168 | 44.4 |  | 1442 | 41.3 |  | 1349 | 52.6 |  | 1294 | 51.0 |
| Tajikistan, 2017 | 57.7 |  | 488 | 61.9 |  | 525 | 46.9 |  | 448 | 35.2 |  | 890 | 56.4 |  | 832 | 58.9 |
| Tanzania, 2015 | 29.6 |  | 572 | 78.8 |  | 613 | 52.1 |  | 537 | 42.1 |  | 1489 | 29.6 |  | 1531 | 29.7 |
| Togo, 2017 | 48.1 |  | 1009 | 44.2 |  | 1102 | 23.7 |  | 909 | 21.0 |  | 726 | 48.1 |  | 735 | 48.1 |
| Uganda, 2016 | 43.8 |  | 1531 | 54.3 |  | 1357 | 40.1 |  | 1272 | 34.7 |  | 2101 | 43.6 |  | 2059 | 43.9 |
| **Lower-middle income countries** |  |  |  |  |  |  |  |  |  |  |  |  |  |  |  |  |
| Armenia, 2015 | 22.4 |  | 175 | 31.9 |  | 178 | 18.0 |  | 146 | 16.1 |  | 260 | 24.9 |  | 239 | 19.8 |
| Bangladesh, 2019 | 45.1 |  | 2177 | 59.9 |  | 2435 | 39.6 |  | 2079 | 35.9 |  | 3439 | 45.7 |  | 3252 | 44.6 |
| Bolivia, 2016 | 19.2 |  | 508 | 32.5 |  | 533 | 13.1 |  | 460 | 11.2 |  | 763 | 19.9 |  | 738 | 18.5 |
| Cameroon, 2018 | 31.8 |  | 884 | 76.3 |  | 984 | 45.0 |  | 800 | 42.2 |  | 1308 | 32.4 |  | 1268 | 31.3 |
| Congo Brazzaville, 2014 | 51.6 |  | 905 | 48.1 |  | 949 | 25.0 |  | 722 | 20.7 |  | 1391 | 53.5 |  | 1374 | 49.8 |
| Cote dIvoire, 2016 | 54.9 |  | 992 | 69.4 |  | 842 | 41.1 |  | 931 | 41.0 |  | 1351 | 56.7 |  | 1317 | 53.2 |
| Egypt, 2014 | 44.7 |  | 1804 | 65.5 |  | 1545 | 39.5 |  | 1485 | 24.4 |  | 2544 | 43.3 |  | 2290 | 46.0 |
| El Salvador, 2014 | 15.9 |  | 904 | 60.7 |  | 823 | 36.8 |  | 858 | 31.9 |  | 1147 | 15.5 |  | 1119 | 16.2 |
| Eswatini, 2014 | 21.4 |  | 1223 | 39.6 |  | 1167 | 21.0 |  | 1119 | 18.6 |  | 398 | 21.9 |  | 391 | 20.8 |
| Ghana, 2017 | 43.3 |  | 346 | 48.2 |  | 344 | 26.8 |  | 344 | 25.9 |  | 1296 | 41.0 |  | 1289 | 45.4 |
| Guatemala, 2014 | 26.8 |  | 1085 | 46.7 |  | 1143 | 32.2 |  | 1009 | 29.9 |  | 1791 | 27.0 |  | 1718 | 26.6 |
| Guyana, 2014 | 33.9 |  | 1670 | 32.5 |  | 1782 | 11.1 |  | 1581 | 12.3 |  | 494 | 34.8 |  | 540 | 32.9 |
| Honduras, 2011 | 36.4 |  | 25199 | 74.7 |  | 24241 | 48.6 |  | 22322 | 39.1 |  | 1700 | 36.6 |  | 1537 | 36.2 |
| India, 2015 | 54.8 |  | 986 | 42.7 |  | 977 | 24.1 |  | 846 | 19.4 |  | 37698 | 54.1 |  | 34064 | 55.3 |
| Indonesia, 2017 | 18.6 |  | 349 | 26.2 |  | 311 | 8.1 |  | 332 | 7.9 |  | 2653 | 16.8 |  | 2380 | 20.2 |
| Kenya, 2014 | 29.0 |  | 216 | 47.2 |  | 258 | 50.5 |  | 195 | 61.6 |  | 1440 | 28.8 |  | 1369 | 29.2 |
| Kiribati, 2018 | 52.6 |  | 1213 | 52.3 |  | 1073 | 30.7 |  | 1142 | 24.8 |  | 353 | 54.4 |  | 316 | 51.1 |
| Kyrgyzstan, 2018 | 14.2 |  | 342 | 50.6 |  | 330 | 30.7 |  | 337 | 32.4 |  | 525 | 16.0 |  | 467 | 12.6 |
| Lao, 2017 | 36.3 |  | 461 | 69.9 |  | 476 | 57.0 |  | 402 | 39.3 |  | 1788 | 36.2 |  | 1640 | 36.4 |
| Lesotho, 2018 | 38.0 |  | 597 | 55.4 |  | 551 | 42.1 |  | 526 | 43.0 |  | 526 | 40.3 |  | 483 | 36.0 |
| Mauritania, 2015 | 51.7 |  | 1053 | 59.7 |  | 1238 | 50.2 |  | 893 | 44.7 |  | 1586 | 49.2 |  | 1598 | 54.3 |
| Mongolia, 2018 | 47.1 |  | 3097 | 69.2 |  | 3321 | 46.4 |  | 2465 | 40.4 |  | 844 | 48.3 |  | 830 | 45.9 |
| Myanmar, 2015 | 55.8 |  | 659 | 51.4 |  | 634 | 37.4 |  | 693 | 29.2 |  | 714 | 59.3 |  | 625 | 52.9 |
| Nepal, 2019 | 39.7 |  | 870 | 79.4 |  | 1027 | 55.1 |  | 669 | 49.9 |  | 1090 | 40.4 |  | 896 | 39.2 |
| Nigeria, 2018 | 52.7 |  | 879 | 21.9 |  | 907 | 9.6 |  | 751 | 12.6 |  | 4555 | 54.4 |  | 4328 | 51.1 |
| Pakistan, 2017 | 61.2 |  | 593 | 39.3 |  | 701 | 24.6 |  | 629 | 23.2 |  | 1318 | 60.0 |  | 1248 | 62.3 |
| Papua New Guinea, 2016 | 15.0 |  | 1423 | 78.5 |  | 1500 | 64.5 |  | 1141 | 60.1 |  | 1327 | 15.4 |  | 1210 | 14.7 |
| Sao Tome and Principe, 2019 | 32.5 |  | 639 | 66.4 |  | 569 | 49.7 |  | 562 | 41.6 |  | 260 | 36.7 |  | 245 | 28.5 |
| Senegal, 2019 | 53.0 |  | 762 | 25.3 |  | 721 | 11.5 |  | 783 | 10.9 |  | 888 | 52.3 |  | 882 | 53.8 |
| State of Palestine, 2019 | 28.6 |  | 156 | 45.3 |  | 209 | 27.2 |  | 140 | 26.2 |  | 1009 | 29.7 |  | 914 | 27.6 |
| Sudan, 2014 | 68.2 |  | 249 | 32.5 |  | 286 | 15.3 |  | 254 | 16.7 |  | 2089 | 67.6 |  | 1975 | 68.7 |
| Timor Leste, 2016 | 35.5 |  | 684 | 59.0 |  | 727 | 26.5 |  | 539 | 18.4 |  | 1009 | 36.7 |  | 941 | 34.3 |
| Tunisia, 2018 | 21.4 |  | 290 | 26.1 |  | 315 | 15.2 |  | 341 | 23.2 |  | 499 | 20.6 |  | 447 | 22.0 |
| Vietnam, 2013 | 13.8 |  | 333 | 27.5 |  | 391 | 11.5 |  | 394 | 4.4 |  | 601 | 15.8 |  | 517 | 12.1 |
| Yemen, 2013 | 66.3 |  | 1536 | 74.9 |  | 1718 | 63.7 |  | 1045 | 57.9 |  | 2226 | 66.0 |  | 2073 | 66.6 |
| Zambia, 2018 | 29.6 |  | 937 | 45.1 |  | 937 | 25.3 |  | 911 | 18.3 |  | 1373 | 29.5 |  | 1412 | 29.8 |
| Zimbabwe, 2019 | 29.0 |  | 584 | 48.1 |  | 580 | 21.9 |  | 573 | 16.9 |  | 876 | 29.9 |  | 861 | 28.2 |
| **Upper-middle income countries** |  |  |  |  |  |  |  |  |  |  |  |  |  |  |  |  |
| Albania, 2017 | 26.0 |  | 1384 | 51.1 |  | 1385 | 32.9 |  | 1240 | 23.5 |  | 382 | 23.6 |  | 384 | 28.4 |
| Algeria, 2018 | 24.8 |  | 250 | 36.4 |  | 269 | 19.0 |  | 247 | 20.9 |  | 2221 | 25.9 |  | 2141 | 23.8 |
| Angola, 2015 | 36.5 |  | 227 | 41.8 |  | 281 | 27.5 |  | 222 | 20.9 |  | 2032 | 38.6 |  | 1977 | 34.4 |
| Belize, 2015 | 30.6 |  | 1766 | 37.4 |  | 1633 | 24.0 |  | 1624 | 22.5 |  | 376 | 29.4 |  | 354 | 31.8 |
| Colombia, 2010 | 28.3 |  | 327 | 14.7 |  | 353 | 6.5 |  | 355 | 11.0 |  | 2594 | 30.4 |  | 2429 | 26.4 |
| Costa Rica, 2018 | 10.5 |  | 509 | 46.9 |  | 577 | 33.8 |  | 542 | 39.5 |  | 524 | 8.5 |  | 511 | 12.6 |
| Cuba, 2019 | 40.5 |  | 2294 | 48.2 |  | 1686 | 30.6 |  | 2255 | 26.6 |  | 845 | 36.7 |  | 783 | 44.2 |
| Dominican Republic, 2014 | 35.5 |  | 1409 | 34.2 |  | 1515 | 24.6 |  | 1438 | 16.1 |  | 3172 | 35.7 |  | 3063 | 35.3 |
| Ecuador, 2012 | 23.7 |  | 1109 | 39.9 |  | 1110 | 14.2 |  | 974 | 13.9 |  | 1658 | 23.5 |  | 1533 | 23.7 |
| Gabon, 2012 | 54.9 |  | 652 | 62.4 |  | 544 | 48.9 |  | 523 | 50.0 |  | 858 | 51.7 |  | 861 | 57.9 |
| Georgia, 2018 | 12.4 |  | 225 | 19.0 |  | 256 | 6.0 |  | 220 | 12.3 |  | 356 | 11.1 |  | 345 | 13.7 |
| Iraq, 2018 | 25.8 |  | 1581 | 33.5 |  | 1674 | 23.0 |  | 1531 | 20.4 |  | 2475 | 25.6 |  | 2311 | 26.0 |
| Jordan, 2017 | 41.2 |  | 1016 | 53.8 |  | 857 | 36.7 |  | 807 | 30.1 |  | 1391 | 42.1 |  | 1289 | 40.5 |
| Kazakhstan, 2015 | 21.2 |  | 529 | 40.9 |  | 571 | 12.8 |  | 532 | 9.7 |  | 846 | 21.1 |  | 786 | 21.3 |
| Kosovo, 2019 | 15.4 |  | 276 | 15.7 |  | 278 | 14.2 |  | 294 | 17.8 |  | 250 | 15.0 |  | 221 | 15.8 |
| Maldives, 2016 | 15.8 |  | 775 | 28.0 |  | 770 | 15.0 |  | 766 | 12.5 |  | 435 | 19.1 |  | 413 | 12.8 |
| Mexico, 2015 | 18.7 |  | 149 | 16.3 |  | 167 | 13.0 |  | 140 | 10.7 |  | 1152 | 18.5 |  | 1159 | 18.9 |
| Montenegro, 2018 | 13.0 |  | 120 | 16.0 |  | 107 | 11.0 |  | 105 | 11.5 |  | 176 | 14.6 |  | 156 | 11.7 |
| Namibia, 2013 | 52.1 |  | 492 | 63.0 |  | 458 | 47.6 |  | 353 | 41.9 |  | 619 | 51.3 |  | 684 | 53.0 |
| North Macedonia, 2018 | 13.3 |  | 2143 | 9.5 |  | 2036 | 5.1 |  | 2010 | 4.7 |  | 233 | 11.2 |  | 223 | 15.3 |
| Paraguay, 2016 | 16.5 |  | 431 | 22.9 |  | 536 | 13.4 |  | 476 | 14.1 |  | 757 | 17.6 |  | 686 | 15.6 |
| Peru, 2019 | 6.4 |  | 186 | 4.0 |  | 181 | 1.9 |  | 203 | 1.6 |  | 3170 | 6.6 |  | 3019 | 6.3 |
| Serbia, 2019 | 2.5 |  | 419 | 49.2 |  | 391 | 31.7 |  | 372 | 34.8 |  | 315 | 2.2 |  | 255 | 2.8 |
| South Africa, 2016 | 37.7 |  | 1246 | 20.3 |  | 1326 | 12.7 |  | 1553 | 12.1 |  | 464 | 36.5 |  | 413 | 38.8 |
| Suriname, 2018 | 39.3 |  | 362 | 21.3 |  | 316 | 10.7 |  | 380 | 4.6 |  | 634 | 39.2 |  | 548 | 39.4 |
| Thailand, 2019 | 14.9 |  | 121 | 14.4 |  | 119 | 6.1 |  | 127 | 14.5 |  | 2124 | 16.5 |  | 2001 | 13.5 |
| Tonga, 2019 | 12.0 |  | 177 | 23.3 |  | 147 | 13.7 |  | 147 | 8.0 |  | 178 | 13.2 |  | 189 | 10.6 |
| Turkmenistan, 2019 | 12.2 |  | 285 | 44.4 |  | 313 | 36.0 |  | 279 | 32.4 |  | 559 | 12.4 |  | 499 | 12.0 |
|  |  |  |  |  |  |  |  |  |  |  |  |  |  |  |  |  |
|  |  |  |  |  |  |  |  |  |  |  |  |  |  |  |  |  |

**Supplementary Table 2:** Prevalence of EFF according to child age group and sex

|  |  |  |  |  |  |  |  |  |  |  |  |  |  |  |  |  |
| --- | --- | --- | --- | --- | --- | --- | --- | --- | --- | --- | --- | --- | --- | --- | --- | --- |
|  |  |  |  |  |  |  |  |  |  |  |  |  |  |  |  |  |
|  | **Any egg or flesh food** | | | | | | | | | | | | | | | |
|  |  |  | **Age groups** | | | | | | | |  | **Child sex** | | | | |
| **Survey** | **Overall** |  | **6-11** | |  | **12-17** | |  | **18-23** | |  | **Girls** | |  | **Boys** | |
|  | **%** |  | **N** | **%** |  | **N** | **%** |  | **N** | **%** |  | **N** | **%** |  | **N** | **%** |
| **Low income countries** |  |  |  |  |  |  |  |  |  |  |  |  |  |  |  |  |
| Afghanistan, 2015 | 30.3 |  | 2627 | 18.6 |  | 3599 | 35.7 |  | 1852 | 37.2 |  | 4152 | 30.7 |  | 3926 | 29.9 |
| Benin, 2017 | 51.9 |  | 1370 | 19.5 |  | 1309 | 26.9 |  | 1179 | 28.8 |  | 1986 | 52.6 |  | 1898 | 51.3 |
| Burkina Faso, 2010 | 22.6 |  | 1482 | 33.4 |  | 1187 | 59.7 |  | 1215 | 67.0 |  | 2092 | 23.2 |  | 2056 | 21.9 |
| Burundi, 2016 | 24.9 |  | 1435 | 11.5 |  | 1445 | 24.8 |  | 1268 | 32.7 |  | 1964 | 23.8 |  | 1894 | 25.9 |
| Central African Republic, 2018 | 23.5 |  | 839 | 18.8 |  | 949 | 27.0 |  | 739 | 24.4 |  | 1260 | 23.6 |  | 1267 | 23.5 |
| Cambodia, 2014 | 82.1 |  | 2248 | 38.1 |  | 2326 | 48.3 |  | 1925 | 55.2 |  | 1073 | 81.4 |  | 1054 | 82.7 |
| Chad, 2019 | 45.6 |  | 315 | 44.1 |  | 311 | 65.6 |  | 243 | 71.1 |  | 2763 | 45.1 |  | 2683 | 46.0 |
| Comoros, 2012 | 59.6 |  | 501 | 19.1 |  | 548 | 25.1 |  | 416 | 28.5 |  | 436 | 61.4 |  | 433 | 57.9 |
| Congo Dem. Republic, 2017 | 46.9 |  | 588 | 16.4 |  | 883 | 32.7 |  | 438 | 50.8 |  | 3294 | 46.8 |  | 3205 | 47.0 |
| Ethiopia, 2019 | 24.2 |  | 829 | 21.2 |  | 1106 | 61.0 |  | 789 | 73.8 |  | 747 | 25.5 |  | 718 | 23.0 |
| Gambia, 2018 | 52.2 |  | 784 | 12.6 |  | 669 | 37.6 |  | 740 | 55.1 |  | 1385 | 51.9 |  | 1339 | 52.5 |
| Guinea, 2018 | 31.9 |  | 556 | 26.3 |  | 625 | 40.8 |  | 471 | 39.9 |  | 987 | 31.9 |  | 922 | 32.0 |
| Guinea Bissau, 2018 | 35.1 |  | 748 | 60.9 |  | 655 | 90.3 |  | 724 | 96.2 |  | 1096 | 38.6 |  | 1097 | 31.6 |
| Haiti, 2016 | 35.6 |  | 558 | 25.6 |  | 511 | 61.9 |  | 454 | 67.2 |  | 832 | 37.7 |  | 820 | 33.5 |
| Liberia, 2019 | 49.6 |  | 1269 | 41.3 |  | 1411 | 51.3 |  | 1179 | 49.2 |  | 759 | 50.3 |  | 764 | 48.9 |
| Madagascar, 2018 | 47.3 |  | 902 | 29.2 |  | 1016 | 52.0 |  | 795 | 62.5 |  | 1962 | 45.7 |  | 1897 | 48.7 |
| Malawi, 2015 | 37.6 |  | 1168 | 30.3 |  | 1157 | 51.6 |  | 955 | 55.9 |  | 2430 | 37.9 |  | 2317 | 37.3 |
| Mali, 2018 | 47.5 |  | 1627 | 26.0 |  | 1627 | 42.5 |  | 1493 | 45.2 |  | 1401 | 48.9 |  | 1312 | 46.3 |
| Mozambique, 2011 | 45.2 |  | 1209 | 8.9 |  | 1251 | 21.7 |  | 800 | 21.0 |  | 1580 | 45.8 |  | 1700 | 44.5 |
| Niger, 2012 | 16.8 |  | 888 | 15.1 |  | 765 | 23.0 |  | 701 | 23.7 |  | 1621 | 16.4 |  | 1639 | 17.2 |
| Rwanda, 2014 | 20.3 |  | 902 | 25.9 |  | 1040 | 63.6 |  | 701 | 74.2 |  | 1175 | 19.8 |  | 1179 | 20.7 |
| Sierra Leone, 2019 | 53.4 |  | 1836 | 36.7 |  | 2168 | 49.4 |  | 1442 | 51.3 |  | 1349 | 54.2 |  | 1294 | 52.6 |
| Tajikistan, 2017 | 37.8 |  | 488 | 41.2 |  | 525 | 64.3 |  | 448 | 77.1 |  | 890 | 36.4 |  | 832 | 39.0 |
| Tanzania, 2015 | 36.1 |  | 572 | 14.2 |  | 613 | 41.8 |  | 537 | 57.4 |  | 1489 | 35.0 |  | 1531 | 37.2 |
| Togo, 2017 | 60.7 |  | 1009 | 24.9 |  | 1102 | 41.9 |  | 909 | 41.3 |  | 726 | 60.2 |  | 735 | 61.3 |
| Uganda, 2016 | 40.6 |  | 1531 | 34.3 |  | 1357 | 42.3 |  | 1272 | 46.8 |  | 2101 | 40.0 |  | 2059 | 41.3 |
| **Lower-middle income countries** |  |  |  |  |  |  |  |  |  |  |  |  |  |  |  |  |
| Armenia, 2015 | 52.3 |  | 175 | 32.1 |  | 178 | 61.6 |  | 146 | 66.2 |  | 260 | 52.9 |  | 239 | 51.7 |
| Bangladesh, 2019 | 60.2 |  | 2177 | 41.4 |  | 2435 | 65.6 |  | 2079 | 73.9 |  | 3439 | 58.9 |  | 3252 | 61.4 |
| Bolivia, 2016 | 82.6 |  | 508 | 64.2 |  | 533 | 90.1 |  | 460 | 94.7 |  | 763 | 84.7 |  | 738 | 80.5 |
| Cameroon, 2018 | 55.5 |  | 884 | 38.0 |  | 984 | 70.6 |  | 800 | 79.7 |  | 1308 | 56.2 |  | 1268 | 54.9 |
| Congo Brazzaville, 2014 | 47.9 |  | 905 | 45.2 |  | 949 | 58.9 |  | 722 | 63.7 |  | 1391 | 44.9 |  | 1374 | 50.8 |
| Cote dIvoire, 2016 | 62.0 |  | 992 | 33.1 |  | 842 | 54.0 |  | 931 | 59.1 |  | 1351 | 60.2 |  | 1317 | 63.9 |
| Egypt, 2014 | 53.1 |  | 1804 | 30.5 |  | 1545 | 60.7 |  | 1485 | 73.3 |  | 2544 | 53.3 |  | 2290 | 52.9 |
| El Salvador, 2014 | 68.1 |  | 904 | 29.4 |  | 823 | 59.4 |  | 858 | 69.1 |  | 1147 | 68.2 |  | 1119 | 67.9 |
| Eswatini, 2014 | 68.9 |  | 1223 | 50.7 |  | 1167 | 73.3 |  | 1119 | 79.4 |  | 398 | 67.3 |  | 391 | 70.5 |
| Ghana, 2017 | 52.5 |  | 346 | 39.9 |  | 344 | 75.0 |  | 344 | 81.0 |  | 1296 | 52.3 |  | 1289 | 52.7 |
| Guatemala, 2014 | 67.2 |  | 1085 | 60.9 |  | 1143 | 80.9 |  | 1009 | 86.6 |  | 1791 | 67.7 |  | 1718 | 66.8 |
| Guyana, 2014 | 64.8 |  | 1670 | 45.6 |  | 1782 | 81.1 |  | 1581 | 87.4 |  | 494 | 65.9 |  | 540 | 63.7 |
| Honduras, 2011 | 76.0 |  | 25199 | 8.4 |  | 24241 | 20.4 |  | 22322 | 25.9 |  | 1700 | 76.6 |  | 1537 | 75.4 |
| India, 2015 | 17.9 |  | 986 | 19.0 |  | 977 | 39.0 |  | 846 | 43.0 |  | 37698 | 18.6 |  | 34064 | 17.3 |
| Indonesia, 2017 | 71.3 |  | 349 | 54.0 |  | 311 | 85.6 |  | 332 | 92.7 |  | 2653 | 72.1 |  | 2380 | 70.6 |
| Kenya, 2014 | 33.3 |  | 216 | 29.2 |  | 258 | 66.2 |  | 195 | 78.5 |  | 1440 | 33.5 |  | 1369 | 33.2 |
| Kiribati, 2018 | 57.8 |  | 1213 | 63.6 |  | 1073 | 85.1 |  | 1142 | 89.3 |  | 353 | 59.0 |  | 316 | 56.8 |
| Kyrgyzstan, 2018 | 77.3 |  | 342 | 29.3 |  | 330 | 44.0 |  | 337 | 45.3 |  | 525 | 77.8 |  | 467 | 76.9 |
| Lao, 2017 | 79.0 |  | 461 | 37.4 |  | 476 | 65.5 |  | 402 | 72.6 |  | 1788 | 77.9 |  | 1640 | 79.9 |
| Lesotho, 2018 | 39.5 |  | 597 | 77.3 |  | 551 | 95.5 |  | 526 | 91.4 |  | 526 | 40.0 |  | 483 | 39.1 |
| Mauritania, 2015 | 47.2 |  | 1053 | 27.0 |  | 1238 | 51.9 |  | 893 | 63.5 |  | 1586 | 49.1 |  | 1598 | 45.3 |
| Mongolia, 2018 | 87.9 |  | 3097 | 28.2 |  | 3321 | 44.7 |  | 2465 | 53.3 |  | 844 | 88.6 |  | 830 | 87.2 |
| Myanmar, 2015 | 58.5 |  | 659 | 30.7 |  | 634 | 41.8 |  | 693 | 47.1 |  | 714 | 57.8 |  | 625 | 59.1 |
| Nepal, 2019 | 39.6 |  | 870 | 26.2 |  | 1027 | 41.0 |  | 669 | 47.2 |  | 1090 | 36.8 |  | 896 | 41.9 |
| Nigeria, 2018 | 41.3 |  | 879 | 38.8 |  | 907 | 41.6 |  | 751 | 42.8 |  | 4555 | 40.7 |  | 4328 | 41.9 |
| Pakistan, 2017 | 38.1 |  | 593 | 43.2 |  | 701 | 73.8 |  | 629 | 76.8 |  | 1318 | 39.4 |  | 1248 | 36.9 |
| Papua New Guinea, 2016 | 40.9 |  | 1423 | 26.4 |  | 1500 | 43.8 |  | 1141 | 49.1 |  | 1327 | 39.1 |  | 1210 | 42.5 |
| Sao Tome and Principe, 2019 | 66.9 |  | 639 | 22.7 |  | 569 | 52.3 |  | 562 | 64.8 |  | 260 | 61.1 |  | 245 | 72.4 |
| Senegal, 2019 | 45.8 |  | 762 | 48.5 |  | 721 | 75.7 |  | 783 | 79.8 |  | 888 | 47.1 |  | 882 | 44.5 |
| State of Palestine, 2019 | 65.5 |  | 156 | 45.8 |  | 209 | 70.9 |  | 140 | 84.1 |  | 1009 | 64.9 |  | 914 | 66.1 |
| Sudan, 2014 | 39.2 |  | 249 | 52.8 |  | 286 | 75.5 |  | 254 | 78.3 |  | 2089 | 39.2 |  | 1975 | 39.2 |
| Timor Leste, 2016 | 46.2 |  | 684 | 29.4 |  | 727 | 52.7 |  | 539 | 58.2 |  | 1009 | 45.8 |  | 941 | 46.6 |
| Tunisia, 2018 | 77.1 |  | 290 | 61.2 |  | 315 | 84.8 |  | 341 | 83.7 |  | 499 | 75.6 |  | 447 | 78.4 |
| Vietnam, 2013 | 89.7 |  | 333 | 76.9 |  | 391 | 93.2 |  | 394 | 97.1 |  | 601 | 90.1 |  | 517 | 89.3 |
| Yemen, 2013 | 34.7 |  | 1536 | 21.4 |  | 1718 | 38.5 |  | 1045 | 48.2 |  | 2226 | 34.6 |  | 2073 | 34.8 |
| Zambia, 2018 | 53.7 |  | 937 | 42.6 |  | 937 | 57.5 |  | 911 | 61.2 |  | 1373 | 53.3 |  | 1412 | 54.1 |
| Zimbabwe, 2019 | 39.5 |  | 584 | 26.5 |  | 580 | 42.3 |  | 573 | 49.8 |  | 876 | 39.3 |  | 861 | 39.7 |
| **Upper-middle income countries** |  |  |  |  |  |  |  |  |  |  |  |  |  |  |  |  |
| Albania, 2017 | 68.5 |  | 1384 | 48.7 |  | 1385 | 63.7 |  | 1240 | 72.2 |  | 382 | 68.4 |  | 384 | 68.5 |
| Algeria, 2018 | 52.9 |  | 250 | 53.3 |  | 269 | 75.5 |  | 247 | 79.3 |  | 2221 | 53.6 |  | 2141 | 52.2 |
| Angola, 2015 | 61.0 |  | 227 | 52.2 |  | 281 | 82.0 |  | 222 | 85.3 |  | 2032 | 63.3 |  | 1977 | 58.7 |
| Belize, 2015 | 72.6 |  | 1766 | 66.7 |  | 1633 | 90.7 |  | 1624 | 91.8 |  | 376 | 72.7 |  | 354 | 72.5 |
| Colombia, 2010 | 82.5 |  | 327 | 57.6 |  | 353 | 88.5 |  | 355 | 91.0 |  | 2594 | 83.8 |  | 2429 | 81.3 |
| Costa Rica, 2018 | 80.7 |  | 509 | 78.5 |  | 577 | 85.2 |  | 542 | 85.0 |  | 524 | 82.8 |  | 511 | 78.5 |
| Cuba, 2019 | 82.6 |  | 2294 | 47.1 |  | 1686 | 76.4 |  | 2255 | 84.1 |  | 845 | 82.9 |  | 783 | 82.4 |
| Dominican Republic, 2014 | 68.6 |  | 1409 | 32.4 |  | 1515 | 57.6 |  | 1438 | 67.6 |  | 3172 | 69.9 |  | 3063 | 67.4 |
| Ecuador, 2012 | 74.8 |  | 1109 | 54.5 |  | 1110 | 86.6 |  | 974 | 87.0 |  | 1658 | 76.0 |  | 1533 | 73.8 |
| Gabon, 2012 | 56.7 |  | 652 | 40.1 |  | 544 | 66.2 |  | 523 | 70.9 |  | 858 | 54.6 |  | 861 | 58.8 |
| Georgia, 2018 | 61.0 |  | 225 | 39.1 |  | 256 | 74.6 |  | 220 | 70.1 |  | 356 | 63.4 |  | 345 | 58.6 |
| Iraq, 2018 | 64.9 |  | 1581 | 46.5 |  | 1674 | 71.9 |  | 1531 | 77.9 |  | 2475 | 63.4 |  | 2311 | 66.4 |
| Jordan, 2017 | 59.6 |  | 1016 | 37.6 |  | 857 | 69.8 |  | 807 | 76.8 |  | 1391 | 56.5 |  | 1289 | 62.3 |
| Kazakhstan, 2015 | 70.8 |  | 529 | 42.8 |  | 571 | 82.2 |  | 532 | 87.6 |  | 846 | 68.7 |  | 786 | 72.7 |
| Kosovo, 2019 | 55.9 |  | 276 | 44.5 |  | 278 | 82.3 |  | 294 | 90.8 |  | 250 | 61.9 |  | 221 | 50.4 |
| Maldives, 2016 | 72.0 |  | 775 | 43.2 |  | 770 | 77.4 |  | 766 | 85.1 |  | 435 | 75.4 |  | 413 | 68.8 |
| Mexico, 2015 | 68.0 |  | 149 | 39.3 |  | 167 | 76.2 |  | 140 | 71.1 |  | 1152 | 73.3 |  | 1159 | 62.3 |
| Montenegro, 2018 | 78.1 |  | 120 | 68.4 |  | 107 | 85.7 |  | 105 | 82.0 |  | 176 | 74.7 |  | 156 | 80.8 |
| Namibia, 2013 | 64.2 |  | 492 | 47.0 |  | 458 | 70.7 |  | 353 | 81.2 |  | 619 | 65.0 |  | 684 | 63.3 |
| North Macedonia, 2018 | 63.4 |  | 2143 | 84.4 |  | 2036 | 94.9 |  | 2010 | 95.6 |  | 233 | 69.4 |  | 223 | 58.0 |
| Paraguay, 2016 | 73.6 |  | 431 | 51.1 |  | 536 | 80.1 |  | 476 | 86.9 |  | 757 | 72.8 |  | 686 | 74.4 |
| Peru, 2019 | 91.6 |  | 186 | 82.7 |  | 181 | 95.9 |  | 203 | 96.0 |  | 3170 | 92.1 |  | 3019 | 91.0 |
| Serbia, 2019 | 91.4 |  | 419 | 21.4 |  | 391 | 52.9 |  | 372 | 67.8 |  | 315 | 90.2 |  | 255 | 92.2 |
| South Africa, 2016 | 61.1 |  | 1246 | 80.0 |  | 1326 | 94.0 |  | 1553 | 95.1 |  | 464 | 63.1 |  | 413 | 59.2 |
| Suriname, 2018 | 45.5 |  | 362 | 48.1 |  | 316 | 71.9 |  | 380 | 82.6 |  | 634 | 45.9 |  | 548 | 45.1 |
| Thailand, 2019 | 90.0 |  | 121 | 62.1 |  | 119 | 76.2 |  | 127 | 88.2 |  | 2124 | 89.2 |  | 2001 | 90.7 |
| Tonga, 2019 | 75.8 |  | 177 | 30.0 |  | 147 | 67.7 |  | 147 | 74.2 |  | 178 | 73.9 |  | 189 | 78.0 |
| Turkmenistan, 2019 | 67.5 |  | 285 | 40.1 |  | 313 | 66.3 |  | 279 | 77.7 |  | 559 | 67.3 |  | 499 | 67.6 |
|  |  |  |  |  |  |  |  |  |  |  |  |  |  |  |  |  |
|  |  |  |  |  |  |  |  |  |  |  |  |  |  |  |  |  |

**Supplementary Table 3:** Slope index of inequalities (SII) and respective 95% confidence interval (CI) for the prevalence of ZVF and EFF according to wealth quintiles

|  | | | | | |  |
| --- | --- | --- | --- | --- | --- | --- |
|  |  |  |  |  |  |  |
|  | **Zero vegetable or fruit** | |  | **Any egg or flesh food** | |  |
| **Survey** |  |  |  |  |  |  |
|  | **SII** | **95% CI** |  | **SII** | **95% CI** |  |
| **Low income countries** |  |  |  |  |  |  |
| Afghanistan, 2015 | -10.0 | -16.7; -3.4 |  | 8.2 | 1.8; 14.6 |  |
| Benin, 2017 | -11.7 | -17.4; -6.0 |  | 23.7 | 18.1; 29.3 |  |
| Burkina Faso, 2010 | -7.2 | -12.4; -2.0 |  | 25.1 | 20.1; 30.0 |  |
| Burundi, 2016 | -7.7 | -12.0; -3.4 |  | 32.1 | 27.0; 37.2 |  |
| Cambodia, 2014 | -14.4 | -23.3; -5.5 |  | 4.1 | -3.6; 11.7 |  |
| Central African Republic, 2018 | -6.1 | -13.6; 1.3 |  | 29.2 | 23.2; 35.2 |  |
| Chad, 2019 | -18.0 | -23.5; -12.5 |  | 18.4 | 13.0; 23.9 |  |
| Comoros, 2012 | -13.8 | -27.2; -0.3 |  | 4.4 | -9.2; 18.1 |  |
| Congo Democratic Republic, 2017 | 16.0 | 9.5; 22.4 |  | 22.0 | 15.2; 28.7 |  |
| Ethiopia, 2019 | -31.7 | -43.0; -20.3 |  | 24.6 | 13.4; 35.8 |  |
| Gambia, 2018 | 2.7 | -5.9; 11.2 |  | -6.0 | -15.0; 3.0 |  |
| Guinea Bissau, 2018 | -12.3 | -20.4; -4.2 |  | 6.1 | -2.3; 14.5 |  |
| Guinea, 2018 | 5.9 | -2.1; 14.0 |  | 29.1 | 21.7; 36.6 |  |
| Haiti, 2016 | -21.0 | -30.7; -11.4 |  | 28.5 | 19.5; 37.5 |  |
| Liberia, 2019 | 8.9 | -3.7; 21.5 |  | -12.9 | -25.6; -0.1 |  |
| Madagascar, 2018 | -19.9 | -25.5; -14.4 |  | 46.9 | 41.3; 52.5 |  |
| Malawi, 2015 | -3.0 | -8.3; 2.3 |  | 30.7 | 25.0; 36.4 |  |
| Mali, 2018 | -10.3 | -17.7; -2.8 |  | 32.7 | 25.8; 39.6 |  |
| Mozambique, 2011 | 4.2 | -2.4; 10.7 |  | -7.5 | -14.5; -0.5 |  |
| Niger, 2012 | -28.8 | -34.5; -23.0 |  | 26.8 | 21.8; 31.7 |  |
| Rwanda, 2014 | -21.3 | -27.7; -15.0 |  | 25.5 | 19.7; 31.4 |  |
| Sierra Leone, 2019 | -5.0 | -12.8; 2.8 |  | -3.4 | -11.2; 4.4 |  |
| Tajikistan, 2017 | -17.8 | -26.6; -8.9 |  | 7.6 | -1.2; 16.4 |  |
| Tanzania, 2015 | -14.6 | -21.1; -8.1 |  | 42.1 | 36.0; 48.3 |  |
| Togo, 2017 | 3.6 | -7.5; 14.8 |  | 10.6 | -0.1; 21.3 |  |
| Uganda, 2016 | 13.8 | 7.7; 19.8 |  | 16.7 | 10.7; 22.8 |  |
| **Lower-middle income countries** |  |  |  |  |  |  |
| Armenia, 2015 | -16.4 | -29.8; -2.9 |  | 3.6 | -13.9; 21.0 |  |
| Bangladesh, 2019 | -14.9 | -19.5; -10.3 |  | 18.7 | 14.2; 23.2 |  |
| Bolivia, 2016 | -24.9 | -33.7; -16.2 |  | 7.4 | -0.5; 15.4 |  |
| Cameroon, 2018 | 0.8 | -6.7; 8.3 |  | 40.4 | 33.3; 47.5 |  |
| Congo Brazzaville, 2014 | 28.4 | 19.4; 37.4 |  | -17.7 | -26.9; -8.4 |  |
| Cote dIvoire, 2016 | -0.4 | -9.2; 8.4 |  | 14.0 | 5.7; 22.2 |  |
| Egypt, 2014 | -7.2 | -12.9; -1.4 |  | 4.3 | -1.5; 10.1 |  |
| El Salvador, 2014 | -15.0 | -21.2; -8.9 |  | 3.6 | -4.9; 12.1 |  |
| Eswatini, 2014 | -17.0 | -27.9; -6.2 |  | 18.7 | 5.9; 31.6 |  |
| Ghana, 2017 | -12.6 | -22.1; -3.1 |  | 30.9 | 22.0; 39.7 |  |
| Guatemala, 2014 | -24.6 | -30.3; -18.9 |  | 28.5 | 22.4; 34.6 |  |
| Guyana, 2014 | -18.7 | -30.7; -6.8 |  | 1.2 | -11.2; 13.6 |  |
| Honduras, 2011 | -31.5 | -37.7; -25.3 |  | 9.6 | 3.6; 15.6 |  |
| India, 2015 | -9.4 | -11.3; -7.6 |  | 5.8 | 4.2; 7.4 |  |
| Indonesia, 2017 | -18.5 | -23.0; -14.0 |  | 19.4 | 14.1; 24.8 |  |
| Kenya, 2014 | -48.8 | -54.7; -42.9 |  | 22.2 | 14.2; 30.1 |  |
| Kiribati, 2018 | -18.9 | -32.2; -5.5 |  | -13.1 | -26.6; 0.3 |  |
| Kyrgyzstan, 2018 | -18.3 | -27.2; -9.4 |  | 5.1 | -5.4; 15.5 |  |
| Lao, 2017 | -25.2 | -31.2; -19.2 |  | 24.5 | 19.1; 29.9 |  |
| Lesotho, 2018 | -8.0 | -20.5; 4.6 |  | 28.2 | 16.2; 40.2 |  |
| Mauritania, 2015 | -67.8 | -72.8; -62.7 |  | 22.2 | 14.5; 29.9 |  |
| Mongolia, 2018 | -62.2 | -70.2; -54.2 |  | -1.4 | -9.2; 6.3 |  |
| Myanmar, 2015 | -22.6 | -33.7; -11.6 |  | 21.8 | 10.9; 32.6 |  |
| Nepal, 2019 | -12.2 | -21.6; -2.8 |  | 27.1 | 17.7; 36.4 |  |
| Nigeria, 2018 | -8.2 | -12.6; -3.9 |  | 58.5 | 55.4; 61.7 |  |
| Pakistan, 2017 | -17.9 | -26.6; -9.2 |  | 35.5 | 27.6; 43.5 |  |
| Papua New Guinea, 2016 | -4.5 | -10.8; 1.7 |  | 37.9 | 28.7; 47.1 |  |
| Sao Tome and Principe, 2019 | -29.7 | -44.4; -15.0 |  | 8.1 | -7.4; 23.5 |  |
| Senegal, 2019 | -8.7 | -19.6; 2.1 |  | 9.0 | -1.9; 19.8 |  |
| State of Palestine, 2019 | -34.0 | -41.6; -26.4 |  | 21.1 | 12.8; 29.4 |  |
| Sudan, 2014 | -33.6 | -39.9; -27.3 |  | 38.3 | 31.9; 44.7 |  |
| Timor Leste, 2016 | -15.2 | -23.7; -6.8 |  | 28.3 | 19.6; 36.9 |  |
| Tunisia, 2018 | -23.9 | -33.5; -14.3 |  | 14.2 | 4.5; 23.9 |  |
| Vietnam, 2013 | -28.9 | -36.7; -21.1 |  | 15.0 | 8.0; 22.0 |  |
| Yemen, 2013 | -42.8 | -48.3; -37.3 |  | 32.9 | 27.0; 38.7 |  |
| Zambia, 2018 | -14.0 | -21.2; -6.9 |  | 40.4 | 33.7; 47.2 |  |
| Zimbabwe, 2019 | 2.9 | -5.4; 11.3 |  | 47.6 | 39.9; 55.2 |  |
| **Upper-middle income countries** |  |  |  |  |  |  |
| Albania, 2017 | -22.9 | -38.8; -7.0 |  | -8.0 | -26.7; 10.6 |  |
| Algeria, 2018 | -19.1 | -24.9; -13.3 |  | 19.8 | 12.9; 26.7 |  |
| Angola, 2015 | 2.1 | -5.0; 9.2 |  | 19.9 | 12.7; 27.1 |  |
| Belize, 2015 | -32.9 | -45.4; -20.3 |  | 4.1 | -10.4; 18.5 |  |
| Colombia, 2010 | -21.4 | -27.2; -15.6 |  | 4.6 | -0.5; 9.7 |  |
| Costa Rica, 2018 | -15.6 | -29.0; -2.2 |  | -3.9 | -21.9; 14.2 |  |
| Cuba, 2019 | -8.1 | -24.3; 8.1 |  | 13.0 | 0.4; 25.6 |  |
| Dominican Republic, 2014 | -14.7 | -20.7; -8.8 |  | 17.0 | 11.3; 22.8 |  |
| Ecuador, 2012 | -10.7 | -20.3; -1.0 |  | -0.4 | -10.5; 9.7 |  |
| Gabon, 2012 | 9.1 | -3.2; 21.4 |  | 1.1 | -11.3; 13.5 |  |
| Georgia, 2018 | -9.6 | -21.2; 1.9 |  | 4.2 | -13.9; 22.3 |  |
| Iraq, 2018 | -15.9 | -22.0; -9.7 |  | 8.8 | 0.7; 16.9 |  |
| Jordan, 2017 | -11.0 | -21.2; -0.9 |  | 8.7 | -1.4; 18.7 |  |
| Kazakhstan, 2015 | -10.6 | -20.5; -0.7 |  | 2.3 | -8.1; 12.7 |  |
| Kosovo, 2019 | -29.5 | -41.9; -17.2 |  | 15.5 | -0.7; 31.7 |  |
| Maldives, 2016 | -11.3 | -23.2; 0.6 |  | -3.0 | -18.9; 12.9 |  |
| Mexico, 2015 | -14.9 | -23.7; -6.1 |  | 11.8 | 0.9; 22.6 |  |
| Montenegro, 2018 | -37.6 | -59.4; -15.8 |  | 39.8 | 13.0; 66.6 |  |
| Namibia, 2013 | -48.3 | -57.1; -39.4 |  | 22.3 | 12.4; 32.3 |  |
| North Macedonia, 2018 | -25.5 | -41.3; -9.6 |  | 35.3 | 16.5; 54.0 |  |
| Paraguay, 2016 | -25.1 | -33.2; -17.1 |  | -4.3 | -15.1; 6.5 |  |
| Peru, 2019 | -8.1 | -10.9; -5.3 |  | 5.6 | 2.2; 8.9 |  |
| Serbia, 2019 | -9.3 | -17.7; -1.0 |  | 9.9 | -1.9; 21.8 |  |
| South Africa, 2016 | -30.1 | -43.0; -17.2 |  | 4.5 | -9.6; 18.6 |  |
| Suriname, 2018 | -25.6 | -38.7; -12.6 |  | 9.6 | -3.6; 22.7 |  |
| Thailand, 2019 | -9.5 | -17.5; -1.4 |  | 1.1 | -5.0; 7.2 |  |
| Tonga, 2019 | -2.4 | -17.1; 12.3 |  | -5.0 | -26.0; 16.1 |  |
| Turkmenistan, 2019 | 6.3 | -0.5; 13.0 |  | -9.0 | -19.5; 1.4 |  |
|  |  |  |  |  |  |  |
|  |  |  |  |  |  |  |

**Supplementary Figure 1.** Prevalence of ZVF according to area of residence (urban or rural)

**Supplementary Figure 2.** Prevalence of EFF according to area of residence (urban or rural)

**Supplementary Figure 3.** Prevalence of ZVF according to wealth quintiles

**Supplementary Figure 4.** Prevalence of EFF according to wealth quintiles
